# Plant–Microbe Interactions as Emerging Biomanufacturing Platforms for Iron Biofortification, with Rice as a Principal Model

**DOI:** 10.3390/life16101742

**Published:** 2026-10-17

**Authors:** Winston Franz Ríos-Ruiz, Marvin Barrera-Lozano, Edson Esmith Torres-Chávez, Henry Giovani Jave-Concepción, Lenin Torres-Bernal, Danny Fran Pompa-Vásquez, Ivone Vásquez-Briones

**Affiliations:** 1 Laboratorio de Microbiología Agrícola “Raúl Ríos Reátegui”, Departamento Académico Agrosilvopastoril, Facultad de Ciencias Agrarias, Universidad Nacional de San Martín, Jr. Amorarca 334, Ciudad Universitaria, Morales 22001, San Martín, Peru; henryjavec@unsm.edu.pe (H.G.J.-C.); df.pompava@unsm.edu.pe (D.F.P.-V.); ivasquezb@alumno.unsm.edu.pe (I.V.-B.); 2 Laboratorio de Botánica y Dendrología, Departamento Académico Agrosilvopastoril, Facultad de Ciencias Agrarias, Universidad Nacional de San Martín, Jr. Amorarca 334, Ciudad Universitaria, Morales 22001, San Martín, Peru; mbarrera@unsm.edu.pe; 3 Centro de Investigación en Semillas AGROSINOR, Av. Hermógenes Mejía Solf Nro. S/n Sector Santa Teresita, Jaén 06800, Cajamarca, Peru; torres_2013_25@hotmail.com; 4 Facultad de Medicina Veterinaria, Universidad Nacional de San Martín, Fundo Miraflores, La Banda de Shilcayo 22200, San Martín, Peru; lenbernal@unsm.edu.pe

**Keywords:** iron biofortification, biomanufacturing platforms, biofabrication, siderophores, PGPR, iron homeostasis, plant microbiome, rice

## Abstract

Iron deficiency remains a major public health challenge in populations relying on staple foods with low iron bioavailability, including polished rice. This narrative review evaluates how beneficial plant-associated microorganisms may support iron (Fe) biofortification by influencing Fe mobilization in the rhizosphere, root acquisition, and subsequent transport to edible tissues. We organize the existing evidence along a soil–plant–grain–food continuum and distinguish measured outcomes from proposed mechanistic links. This evidence-based framework is described as a plant–microbe biomanufacturing platform in a strictly conceptual sense: neither plants nor microorganisms manufacture Fe; rather, their interactions can modify its mobilization and distribution. Most studies have demonstrated microbial effects on Fe availability and plant physiology, but fewer have established increased Fe in grain, and evidence for Fe being retained after polishing or absorbed by consumers remains scarce. Future work should integrate field validation, nitrogen and water management, Fe localization, food processing, and nutritional bioavailability. This approach may help prioritize testable interventions without equating microbial activity or higher total grain Fe with improved human iron status.

## 1. Introduction

### 1.1. Iron Deficiency and Food Security

Iron deficiency is one of the most common micronutrient deficiencies worldwide and disproportionately affects children, women of reproductive age, and populations in low- and middle-income countries [1]. Iron is essential for oxygen transport, energy metabolism, and multiple cellular processes; its homeostasis depends on mineral absorption, transport, storage, and recycling [2,3]. Low intake and bioavailability of iron are important contributors to iron deficiency and anemia, particularly in diets based predominantly on plant foods [4]. Despite the interventions implemented, anemia continues to be a significant public health problem. The World Health Organization reported that the global prevalence of anemia in women aged 15 to 49 years increased from 27.6% in 2012 to 30.7% in 2023, affecting approximately 605 million women [5]. Iron deficiency during critical stages of development can affect brain function, while in school-aged children, iron supplementation has shown favorable effects on certain cognitive domains, especially when a prior deficiency exists [6,7]. In this scenario, improving the contribution and bioavailability of iron from staple foods constitutes an important component of food and nutritional security strategies, complementary to conventional supplementation and fortification [8,9].

### 1.2. Iron Biofortification: Progress and Limitations

Biofortification aims to increase the micronutrient density in the edible parts of crops through genetic improvement, agronomic practices, or biotechnological tools. Unlike post-harvest fortification, enrichment occurs during crop development and production, which can make it more accessible to populations that depend on staple foods [10,11,12]. Recent studies in rice also confirm that agronomic biofortification practices can modify the concentration of Fe and other micronutrients in the grain, although the response depends on the application method and dose [13]. Conventional genetic improvement and genomic tools allow the identification of loci, candidate genes, and mechanisms related to micronutrient accumulation. In cereals, the evidence is particularly extensive for wheat and rice, with significant advances in identifying loci, candidate genes, and mechanisms related to Fe and Zn accumulation in the grain [14,15,16]. These advances provide a basis for combining selection, molecular improvement, and biotechnology, while simultaneously improving yield, agronomic stability, and nutritional quality. Agronomic biofortification involves the application of fertilizers or micronutrient sources to the soil, seed, or foliage. Recent literature shows that these strategies can increase iron (Fe) concentrations in edible tissues and organs, but their effectiveness depends on the species, nutrient source and dosage, application route, and soil and crop properties [17,18,19]. In rice, the low availability of Fe in certain soils and the regulation of its absorption, transport, and partitioning within the plant limit the effective transfer of nutrients to the grain [20,21]. In addition to total Fe content, bioavailability is a key limitation. Components of the food matrix, including phytates and certain phenolic compounds, can reduce the fraction of non-heme iron available for absorption; therefore, a higher analytical concentration of the mineral does not necessarily imply a proportional nutritional benefit [4,22,23]. Consequently, biofortification strategies must consider the micronutrient concentration, its location in the edible tissue, and the factors that influence its utilization by the organism. The adoption of biofortified crops can also be conditioned by socioeconomic factors, seed and input availability, consumer acceptance, information, regulatory frameworks, and the need for validation in local contexts [24,25]. These limitations justify the investigation of complementary approaches capable of harnessing biological processes in the rhizosphere. In this context, plant growth-promoting microorganisms can improve nutrient acquisition and crop performance; however, their effect on micronutrient concentration in the edible fraction must be demonstrated specifically for each plant–microorganism–environment interaction [26,27].

### 1.3. Plants as Emerging Biomanufacturing Platforms

In its conventional application, plant-based biomanufacturing primarily involves the production of recombinant proteins and other valuable biological products through plant molecular farming, integrating molecular engineering, cultivation, downstream processing, and quality control [28]. In contrast, the framework proposed in this review does not imply the synthesis of elemental Fe or the discovery of a new Fe-acquisition mechanism. Instead, it provides an integrative and evaluative perspective on established plant–microbe interactions, encompassing microbial Fe mobilization, root acquisition, internal translocation, grain accumulation, retention after processing, and nutritional utilization. These successive stages represent distinct, experimentally testable processes, and improvements at one stage do not necessarily translate into enhanced Fe accumulation or nutritional benefits at subsequent stages.

In this review, microbiome-assisted Fe biofortification refers to interventions involving plant-associated microorganisms aimed at increasing Fe accumulation in edible crop tissues. The term “plant–microbe biomanufacturing platform” is used exclusively as a conceptual analogy to describe the coordination of soil–microbe–plant processes, rather than as a new biological mechanism or an alternative to established biofortification approaches. Similarly, “biofabrication” is used descriptively to denote the operational integration of microbial Fe mobilization with plant Fe acquisition, transport, and partitioning. Building on the established concepts of PGPR-mediated biofortification and rhizosphere engineering [27,29,30], the proposed framework seeks to distinguish experimentally demonstrated effects from hypothesized mechanisms, identify critical knowledge gaps, and guide the selection of appropriate endpoints for evaluating Fe biofortification and its nutritional relevance.

Accordingly, “microbiome-assisted Fe biofortification” is retained as the preferred term for the intervention throughout this review, whereas “biomanufacturing platform” refers specifically to the integrative conceptual framework and “biofabrication” refers to the operational coordination of the processes involved. This terminology is intended to improve conceptual clarity without overstating the novelty of the underlying biological mechanisms.

### 1.4. Scope and Objective of the Review

The aim of this review is to critically analyze how plant–microorganism interactions can function as emerging biomanufacturing platforms for iron biofortification. Within this framework, biofabrication is examined as the operational concept through which microbial functions and plant physiological processes are intentionally coordinated to improve iron mobilization, acquisition, transport, and partitioning. We examine iron dynamics in the soil–plant system, plant mechanisms of acquisition and homeostasis, the contribution of plant growth–promoting rhizobacteria (PGPR) and siderophores, and the available evidence on iron transfer to edible tissues, with a particular focus on rice. We also analyze integration with nitrogen fertilization, technological bottlenecks, and prospects for scaling up, formulation, and nutritional evaluation.

## 2. Materials and Methods

An integrative narrative review of the scientific literature was conducted. The database searches covered publication records available through 31 August 2026 in the Web of Science Core Collection, Scopus, ScienceDirect, and Google Scholar. The core Boolean strategy combined (“iron” OR “Fe”) AND (“biofortification” OR “grain iron” OR “iron accumulation”) AND (“plant growth-promoting rhizobacteria” OR PGPR OR bacteria OR microbiome OR siderophore*) AND (plant* OR crop* OR rice OR “Oryza sativa”). Complementary searches combined (rice OR “*Oryza sativa*”) with (redox OR “iron plaque” OR “radial oxygen loss” OR flooding OR “alternate wetting and drying” OR “iron toxicity”), and searches on conceptual framing used (biomanufacturing OR biofabrication OR “microbiome-assisted biofortification”) AND plant*. Reference lists of key reviews and eligible primary articles were also examined for additional studies. The searches were not restricted by publication year; English-language peer-reviewed literature was prioritized, with emphasis on recent evidence and seminal mechanistic studies.

Records were screened first by title and abstract, and subsequently by full-text review if they potentially addressed at least one step of the soil–rhizosphere–plant–grain–food continuum. Primary studies were eligible for the structured evidence synthesis when they tested a microbial inoculant, microbial consortium, or microbially derived siderophore and reported at least one of the following outcomes: siderophore production or Fe solubilization; plant growth or yield; Fe in roots, shoots, or other vegetative tissues; Fe in grain/seed; Fe in polished grain or endosperm; Fe bioaccessibility; or Fe bioavailability/nutritional efficacy. Studies reporting only general growth promotion without an Fe-related measurement were retained only as contextual evidence and were not classified as direct evidence of Fe biofortification. Reviews were used for conceptual and mechanistic background but were excluded from the primary-study evidence table. Duplicate reports, non-peer-reviewed reports lacking sufficient methodological detail, and studies in which Fe-related outcomes could not be separated from other treatments were also excluded.

Because this is an integrative narrative review, neither a pooled effect size calculation nor a formal risk-of-bias meta-analysis was performed. The 11 studies in Table 1 were purposively selected as illustrative examples spanning distinct evidence levels, not as an exhaustive or quantitatively representative sample. They are grouped by the endpoint actually demonstrated: (A) direct grain/seed Fe enrichment associated with microbial inoculation or microbially derived siderophores; (B) mechanistic evidence for Fe acquisition or plant Fe nutrition without confirmed edible-grain enrichment; and (C) agronomic/environmental context without direct microbially mediated grain Fe evidence. Studies of reduced nitrogen and water/redox management are included only as contextual comparators, not as demonstrations of microbial Fe biofortification. Classification was based on reported endpoints rather than presumed causal pathways; the heterogeneity of crops, interventions, and measurements precludes quantitative comparison across categories.

The searches were not conducted with a prospectively registered systematic-review protocol, and a complete deduplicated search log was not retained. Consequently, reliable counts of initially identified records, duplicates, title/abstract exclusions, and full-text exclusions cannot be reconstructed from the available documentation. A PRISMA-style flow diagram would imply a level of screening traceability that this narrative review cannot substantiate. The 11 selected primary studies are illustrative examples, not the complete number of publications consulted. This limitation is explicitly acknowledged; a reproducible systematic search and screening log are priorities for future evidence synthesis.

In Table 1, NR denotes information not reported or not directly evaluated in the cited study; missing endpoints were not inferred.

Evidence appraisal was qualitative and endpoint-specific. Direct attribution of increased grain Fe to microbial activity is stronger when studies include appropriate uninoculated controls, verified inoculant identity and persistence, defined Fe inputs, and comparable yields. Total grain Fe alone does not distinguish enhanced uptake from altered grain partitioning, concentration dilution, or contamination during sample preparation. Studies reporting only siderophore activity, vegetative Fe, or yield were therefore not treated as direct evidence of nutritional Fe biofortification. These criteria function as interpretive safeguards rather than a validated numerical risk-of-bias scale.

## 3. Iron Dynamics in Soil–Plant Systems

### 3.1. Soil Fe Chemistry

Iron is one of the most abundant elements in the Earth’s crust and in many soils, but its availability to plants is often limited by its chemical speciation. Under aerobic conditions and at a neutral or alkaline pH, Fe^3+^ predominates, forming oxides and hydroxides with low solubility; in contrast, Fe^2+^ is more soluble and can increase under reducing conditions [42,43,44]. This difference between the total abundance and the bioavailable fraction explains why iron deficiency can occur even in soils with high total iron content. Iron availability is controlled by pH, redox potential, organic matter, mineralogy, and interactions with other elements. In calcareous or alkaline soils, precipitation and adsorption reduce its solubility; in flooded soils, the decrease in redox potential favors the reduction in Fe^3+^ to Fe^2+^ and can increase its concentration in the soil solution [45,46]. Organic matter and ligands of plant or microbial origin also modify iron mobility through complexation, reduction, and local pH changes. Therefore, the rhizosphere constitutes a particularly dynamic microenvironment, where root exudation and microorganisms can alter the speciation and availability of Fe compared to non-rhizospheric soil [43,47,48].

### 3.2. Plant Fe Acquisition

Faced with low iron availability, plants have developed two main acquisition strategies, traditionally called Strategy I and Strategy II [20,44,49]. Strategy I, characteristic of dicotyledons and non-grass monocotyledons, involves rhizosphere acidification through H^+^ extrusion, the reduction in Fe^3+^ to Fe^2+^ by membrane ferric reductases, and subsequent Fe^2+^ uptake via specific transporters. The release of phenolic compounds and other metabolites can complement this mechanism, especially under iron deficiency or high pH [20,50]. Strategy II is characteristic of grasses and is based on the synthesis and secretion of phytosiderophores of the mugineic acid family, which chelate Fe^3+^ and allow the uptake of Fe (III)–phytosiderophore complexes via specific transporters. Rice exhibits a unique physiological characteristic: in addition to this system, it can absorb Fe^2+^ directly, a relevant adaptation in flooded soils where the ferrous form is more abundant [21,50]. These strategies constitute the physiological basis upon which both agronomic management and rhizosphere microorganisms act. Understanding their regulation is essential for designing interventions that increase Fe acquisition efficiency without negatively altering the homeostasis of other nutrients [51,52].

### 3.3. Transport, Remobilization, and Storage

Once absorbed by the root, iron must be chelated and transported to prevent precipitation or reactivity. In the xylem, it is primarily mobilized in association with organic ligands, including citrate, while the phloem participates in its redistribution to young tissues, reproductive organs, and seeds [53,54]. Iron homeostasis requires a balance between metabolic availability and the prevention of toxicity, since free iron can catalyze the formation of reactive oxygen species. Proteins such as ferritin allow for relatively safe iron storage and release when metabolic demand requires it, while vacuoles, chelating agents, and transporters contribute to its cellular compartmentalization [55]. In rice, iron accumulation in the grain depends on its remobilization from vegetative tissues, phloem transport, the action of chelating agents and transporters, and deposition in specific compartments. A significant proportion is located in the embryo and outer layers, which are partially removed during polishing, while the endosperm contains lower concentrations [16,56]. This distribution makes the partitioning of Fe to the endosperm a central objective of rice biofortification [16].

## 4. Plant–Microbe Interactions in Iron Acquisition

### 4.1. PGPR Mechanisms

Plant growth-promoting rhizobacteria (PGPR) constitute a diverse functional group of beneficial bacteria associated primarily with the rhizosphere and root surface that can promote plant growth through direct and indirect mechanisms. These include biological nitrogen fixation, nutrient solubilization or mobilization, the production of phytohormones and siderophores, the modulation of root development, and the induction of responses to biotic and abiotic stresses [26,57,58,59,60,61]. Nutrient mobilization is particularly relevant in soils where a high proportion of phosphorus (P), iron (Fe), or zinc (Zn) is in poorly available forms. PGPR can modify these fractions through acidification and the secretion of organic acids, enzymes, and chelating compounds. In the case of iron, siderophore production is especially important under aerobic conditions, where Fe^3+^ has very low solubility [60,62]. Many PGPR also produce or modulate phytohormones such as auxins, gibberellins, and cytokinins. These compounds can modify root architecture and increase soil exploration, generating an indirect effect on water and nutrient uptake. Therefore, a positive response to inoculation should not be attributed solely to Fe solubilization, but rather to the combination of functions expressed by the strain and the host plant [63,64]. Another mechanism is induced systemic resistance (ISR), whereby certain beneficial microorganisms prepare or activate plant defense responses, which is frequently associated with jasmonate and ethylene pathways [65,66]. Although ISR is not a direct biofortification mechanism, it can contribute to maintaining the crop’s physiological state, thereby indirectly influencing its nutritional performance.

### 4.2. Microbial Siderophores

Siderophores are low-molecular-weight metabolites produced primarily by microorganisms under iron limitation. Their high affinity for Fe^3+^ allows them to chelate the metal and form soluble complexes, increasing their mobility in microenvironments where inorganic iron is scarce [67,68,69]. Based on the functional groups that coordinate the metal, siderophores are mainly classified as catecholates, hydroxamates, carboxylates, and mixed structures. Their affinity and stability constants vary widely, which determines their ability to compete for Fe^3+^ and their behavior under different rhizospheric chemical conditions [67,70]. After the formation of the siderophore–Fe^3+^ complex, microorganisms can retrieve it using specific receptors and transport systems. For the plant, this effect can be beneficial when microbial chelation increases the soluble fraction of Fe and facilitates its subsequent transfer or release near the root; however, the microorganisms also compete for the metal. Therefore, the net result depends on the siderophore, strain, plant species, and soil conditions [49,71].

### 4.3. Siderophore–Plant Interactions

The proposed direct uptake of intact microbial Fe–siderophore complexes (sometimes termed Strategy III) should be distinguished from the established Strategy I and Strategy II pathways. Evidence for the uptake of specific complexes in particular experimental systems cannot be generalized to rice grain enrichment. Demonstrating its contribution would require ligand-specific transport evidence, isotope tracing of microbially mobilized Fe, and quantification of Fe delivered to brown and polished grain under controlled and field conditions.

**Figure 1:**
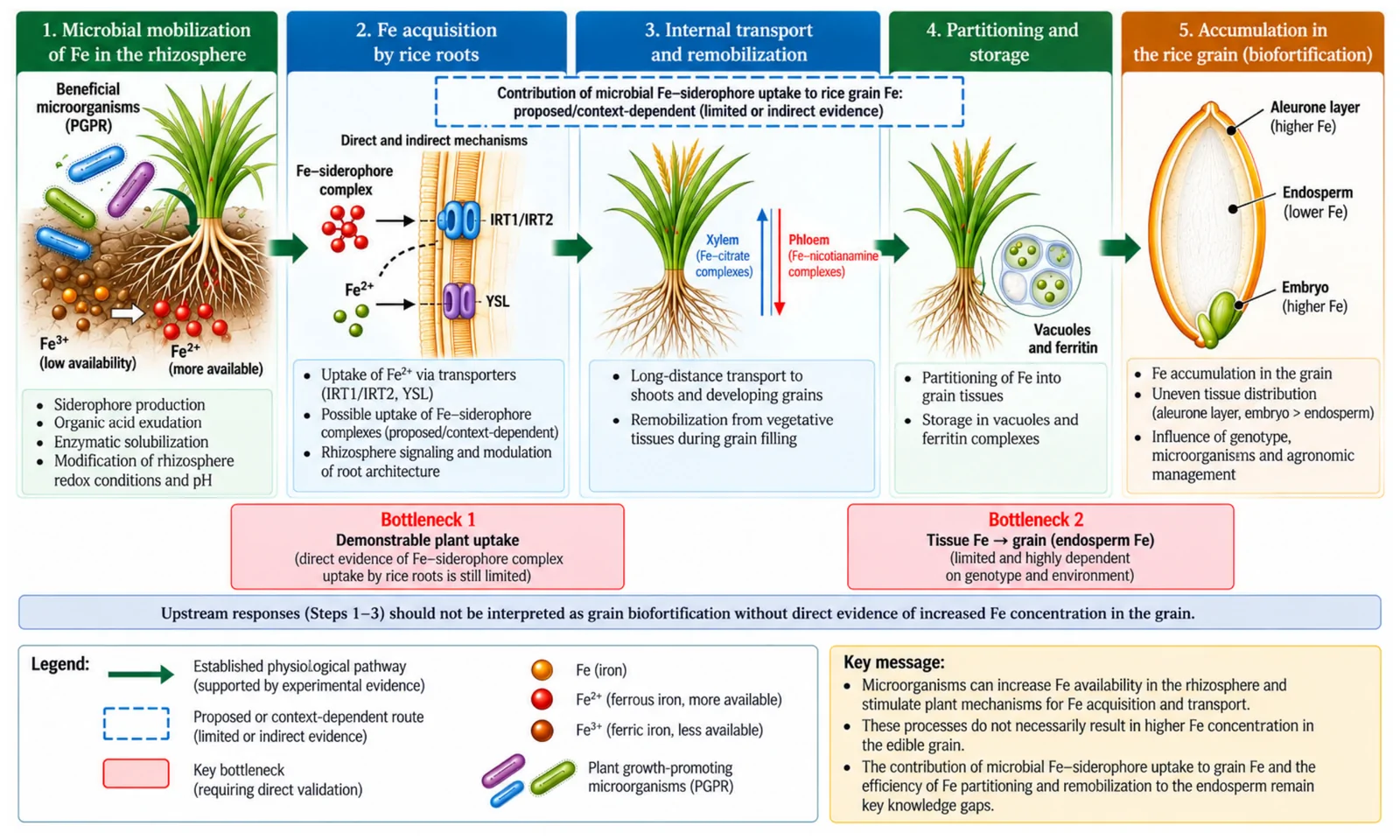
Evidence-based scheme of plant–microbe processes relevant to Fe accumulation in rice. The scheme illustrates five sequential stages: (1) microbial mobilization of Fe in the rhizosphere, including siderophore production and other mechanisms that increase Fe availability; (2) Fe acquisition by rice roots; (3) internal transport and remobilization through xylem and phloem; (4) Fe partitioning and storage in plant tissues; and (5) Fe accumulation in the rice grain. Solid arrows indicate established physiological pathways supported by experimental evidence [20,21,53,56]. The dashed blue box identifies the proposed or context-dependent contribution of microbial Fe–siderophore uptake to rice grain Fe, for which evidence remains limited or indirect. Microbial siderophore-mediated Fe mobilization is experimentally supported, whereas the quantitative contribution of direct microbial Fe–siderophore uptake to Fe accumulation in rice grain under field conditions remains insufficiently established [49]. Two major bottlenecks remain unresolved: demonstrating plant uptake of microbially mobilized Fe (Bottleneck 1) and the subsequent transfer and partitioning of tissue Fe into the grain, particularly the endosperm (Bottleneck 2). Therefore, upstream responses, including increased rhizospheric Fe availability or plant Fe acquisition, should not be interpreted as evidence of grain biofortification without direct demonstration of increased Fe concentration in the edible grain. IRT: iron-regulated transporter; YSL: Yellow Stripe-Like transporter; PGPR: plant growth-promoting rhizobacteria.

The relationship between microbial siderophores and plant nutrition does not necessarily imply that the plant absorbs the entire siderophore–Fe complex intact. In many systems, the benefit derives from the increased solubilization and mobilization of Fe in the rhizosphere, followed by plant mechanisms of reduction, ligand exchange, or uptake via the plant’s own transporters. The nature of these pathways varies among species and continues to be the subject of research [49,72]. In Strategy I plants, Fe mobilized by microbial ligands can become available for reduction processes on the root surface and subsequent uptake as Fe^2+^. In grasses, the interaction may also involve plant phytosiderophores and specific transport systems. Mechanisms for more direct utilization of certain microbial complexes have been proposed, but their physiological importance cannot be generalized to all plant–microorganism associations [49,73]. These interactions occur within a broader chemical dialog: root exudates select and modulate microorganisms, while microbial metabolites alter nutrient availability and plant responses. In rice, Figure 1 organizes this continuum into five mechanistic stages: microbial mobilization of Fe in the rhizosphere; Fe acquisition by roots; internal transport and remobilization; partitioning and storage; and final accumulation in the grain. The figure also distinguishes established physiological pathways from the proposed/context-dependent contribution of microbial Fe–siderophore uptake to rice grain Fe and highlights two major bottlenecks: demonstrable plant uptake and subsequent partitioning of tissue Fe into the grain, particularly the endosperm. The efficiency of each stage depends on the interaction among the microorganism, the plant genotype, and the environment [74,75].

The traditional classification of plant iron nutrition into Strategies I and II may not capture the full diversity of Fe acquisition mechanisms. Recent evidence has led to the proposal of Strategy III, defined as plants directly taking up intact microbial Fe–siderophore complexes, with several potential uptake routes and emerging mechanistic support [49]. However, the prevalence of this mechanism among crops and, critically, its quantitative contribution to Fe accumulation in edible rice grain under field conditions remain to be established. This expanded framework is particularly relevant for evaluating the potential of microbial siderophores in biofortification.

## 5. Plant–Microbe Biomanufacturing Platforms for Iron Biofortification

### 5.1. Conceptual Framework

Critical interpretation of Group A requires caution: the presence of a microbial treatment and higher grain Fe do not by themselves establish the fraction of grain Fe causally attributable to microbial siderophores. The most persuasive experiments compare inoculated and uninoculated plants at an equivalent Fe supply, report both grain Fe concentration and Fe mass per plant or area, document strain establishment, and distinguish brown from polished grain. Stable-isotope tracing and direct assessment of Fe localization would strengthen mechanistic attribution. Cross-crop results cannot be assumed to transfer quantitatively to rice.

**Figure 2:**
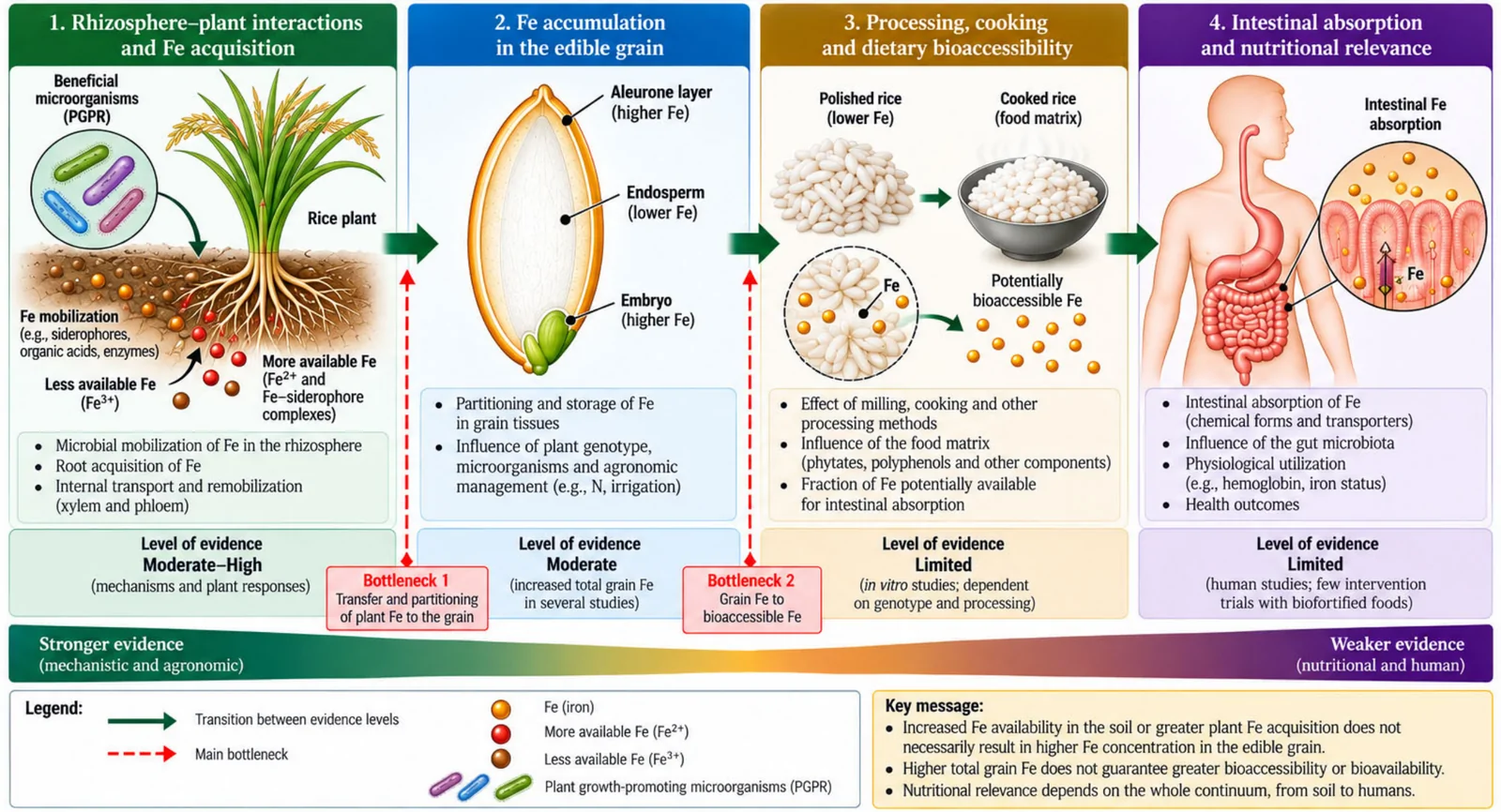
Evidence continuum from microbially assisted Fe acquisition to nutritional relevance. The framework summarizes four sequential evidence levels: (1) rhizosphere–plant Fe interactions, encompassing microbial Fe mobilization, root acquisition, and internal transport; (2) Fe accumulation in the edible grain, representing direct evidence of compositional grain biofortification; (3) processing, cooking, and dietary bioaccessibility, which determine the fraction of grain Fe potentially available for intestinal absorption; and (4) intestinal absorption and nutritional relevance, representing bioavailability and subsequent physiological utilization. Solid arrows represent transitions between evidence levels and should not be interpreted as proof that an upstream response necessarily produces the downstream outcome. The red dashed indicators identify the two main translational bottlenecks: plant/tissue Fe to edible-grain Fe and grain Fe to bioavailable Fe. In particular, increased Fe mobilization or plant Fe acquisition does not necessarily result in higher Fe concentration in the edible grain, and increased total grain Fe does not necessarily imply greater bioaccessibility or bioavailability.

The proposed plant–microbe biomanufacturing framework is a conceptual evidence map, not a novel Fe-acquisition mechanism. Its added explanatory value lies in explicitly distinguishing the microbial mobilization, plant uptake, grain deposition, and nutritional utilization endpoints that are often conflated in discussions of biofortification. Its testable implication is that an intervention cannot be considered successful at a downstream stage unless that stage is directly measured; studies can therefore be designed around the earliest missing link (e.g., isotope-traced uptake, polished-grain Fe, or absorption assays). Figure 1 illustrates the physiological steps and mechanistic uncertainties, whereas Figure 2 presents the corresponding evidence thresholds from soil to nutritional relevance [27,49,76,77].

### 5.2. Evidence Across the Soil-to-Food Continuum

For critical appraisal, microbial Fe mobilization, accumulation in vegetative tissues, concentration in harvested grain, retention after processing, in vitro bioaccessibility, and in vivo bioavailability must be treated as distinct endpoints. The presence of an upstream response cannot establish any downstream outcome. Evidence strength also depends on the presence of appropriate uninoculated controls, identification of the microbial strain and delivery method, experimental replication, environmental context, and measurement of both Fe concentration and yield. Observational or agronomic comparator studies should not be interpreted as direct evidence of microbial Fe biofortification.

Table 1 separates Group A (direct grain/seed Fe outcomes) [31,32,33,34,35,36], Group B (mechanistic or supporting evidence) [37,38], and Group C (agronomic/environmental comparators) [39,40,41]. The groups describe the level and relevance of the reported evidence, not the methodological quality or effect magnitude. In particular, reduced-N growth responses and water-management effects do not demonstrate microbial Fe biofortification. Even Group A generally lacks evidence on polished endosperm Fe, bioaccessibility, or human nutritional efficacy. This classification exposes the measurements needed to test each proposed transition along the soil–plant–food continuum.

Mechanistic studies support microbial modulation of Fe acquisition but do not establish grain enrichment unless grain Fe is measured. In rice, induction of phytosiderophore pathways and Fe-acquisition genes [37] provides physiological support; field studies measuring grain Fe after PGPR inoculation provide stronger, crop-specific evidence [31,32]. Observations in other crops are informative but cannot be assumed to transfer quantitatively to rice [33,34,35,36,38,78,79].

The key translational evidence gap lies beyond total harvested-grain Fe. Studies reporting increased grain or seed Fe [31,32,33,34,35,36] have rarely determined retention in polished rice, Fe speciation, food-matrix inhibitors, or intestinal absorption. Accordingly, grain Fe is treated as compositional evidence, not proof of nutritional efficacy. Future studies should report grain processing state, Fe localization, bioaccessibility, and—where feasible—bioavailability [16,56].

### 5.3. Translation from Grain Fe to Nutritional Relevance

The use of siderophore-producing microorganisms can increase iron availability in the rhizosphere and promote its uptake by the plant; however, the increase in iron in edible tissues also depends on the crop’s translocation and storage capacity. Recent reviews on microbial biofortification highlight that growth-promoting microorganisms can modify the acquisition and assimilation of micronutrients, and, under certain conditions, contribute to increasing their concentration in harvestable organs [27,79]. From a nutritional standpoint, the total iron content is not sufficient to estimate the benefit of a biofortified food. Its bioavailability depends on the chemical form of the element and the presence of dietary components that can inhibit or promote its absorption, including phytates, polyphenols, and ascorbic acid [4,22]. Therefore, although microbial intervention can promote iron nutrition in the plant, further evidence is still needed to establish the extent to which it modifies iron speciation in the grain and its bioavailability to the consumer [27]. The location of iron within the grain is another critical aspect. In cereals, and particularly in rice, a significant proportion of Fe is concentrated in the embryo and outer layers of the grain, while the endosperm contains lower concentrations; consequently, polishing can considerably reduce the final content of this micronutrient [16,56]. Biofortification strategies with the greatest potential nutritional impact should therefore promote not only a greater total accumulation of Fe, but also its redistribution to the endosperm and other fractions that remain after processing.

### 5.4. Current Bottlenecks

The principal bottlenecks for plant–microbe biomanufacturing and its operational biofabrication strategies include context-dependent inoculant performance and establishment within native microbial communities [74,75,80,81], formulation and delivery stability [82], and remaining mechanistic gaps in linking rhizospheric Fe mobilization with Fe acquisition and its subsequent accumulation in edible organs [27,83]. Because these challenges are developed in detail in Section 8, they are introduced here only as constraints that must be resolved before this approach can be translated into reproducible field-scale biofortification strategies.

## 6. Rice as a Model System

### 6.1. Importance

Rice is the principal model in the literature because of its importance as a staple food, the substantial removal of Fe-rich outer grain tissues during polishing, and the strong influence of flooding and root-zone redox gradients on Fe chemistry. Findings from wheat, maize, soybean, quinoa, and sorghum are used as comparative evidence of possible mechanisms, not as proof that identical responses occur in rice.

Rice (*Oryza sativa* L.) is one of the world’s major food crops and a staple food for a substantial proportion of the global population. Its high dietary importance, especially in Asia and large parts of Africa and Latin America, makes any improvement in its nutritional quality a potentially effective food security intervention [16,84]. In populations heavily dependent on rice, this cereal can provide a considerable fraction of daily energy intake; however, polished rice has low concentrations of micronutrients such as iron and zinc. This combination of high consumption and low micronutrient density explains why rice is a priority target for biofortification programs [56,84]. Rice also represents a particularly useful system for studying and developing biofortification strategies due to the wide availability of genomic resources, genes, and loci associated with micronutrient homeostasis, diverse germplasm, and molecular improvement and gene-editing tools. These advances allow for intervention on the absorption, transport, redistribution, and storage processes of iron and zinc in the grain [16,84].

### 6.2. Fe Distribution and Processing

Rice cultivation creates unusually steep and dynamic Fe gradients. Under continuous flooding, microbial respiration lowers soil redox potential and promotes reduction in Fe^3+^ minerals to soluble Fe^2+^; in contrast, oxygen transported through rice aerenchyma leaks radially from roots and creates a comparatively oxidized microsite immediately adjacent to the root. This juxtaposition generates steep redox gradients and favors oxidation of Fe^2+^ to Fe^3+^ oxyhydroxides on the root surface, forming the characteristic Fe plaque [21,46,85,86]. The plaque can act as both a sink and a potentially remobilizable reservoir for Fe and other elements, and its amount and mineralogy change with plant age, pore-water Fe^2+^, and irrigation regime [85,86]. Consequently, rhizospheric Fe concentration alone is an incomplete predictor of Fe entering the plant.

Water management is therefore a central modifier of microbial Fe biofortification in rice. Continuous flooding maintains reducing conditions and generally favors high Fe^2+^ activity, whereas alternate wetting and drying (AWD) periodically re-oxidizes the rhizosphere, modifies Fe-plaque formation and mineralogy, and can alter grain elemental composition. Field evidence indicates that effects of intermittent irrigation regimes on grain Fe are context-dependent: AWD decreased grain Fe overall in one multi-site study, with a significant site × treatment interaction [41], whereas alternate wetting and moderate soil drying also reduced grain Fe in another field study [87]. Excessive Fe^2+^ availability under strongly reducing conditions can also contribute to Fe toxicity in susceptible rice genotypes [88]. Thus, inoculant performance should be evaluated under explicitly defined irrigation/redox regimes rather than extrapolated across flooded and intermittently aerated systems.

In rice, the distinction between mechanistic and compositional evidence is particularly important: beneficial microorganisms can induce Fe-acquisition responses [37], and selected field inoculants have increased grain Fe [31,32]. By contrast, experiments with Bacillus/Priestia under reduced nitrogen [39,40] supported agronomic compatibility but did not quantify grain Fe. These findings should be evaluated under specific irrigation and redox conditions.

Finally, grain Fe concentration must be interpreted together with yield. Treatments that strongly increase biomass or grain yield can produce an apparent concentration dilution when Fe accumulation does not increase proportionally; conversely, a higher concentration can occur without a greater total Fe yield per area. Yield dilution has been identified as a plausible factor when interpreting Fe concentrations in field-grown biofortified rice [89]. Accordingly, rice studies should report both Fe concentration (mg kg^−1^) and total Fe accumulation or Fe yield per plant/area, together with grain yield and the processed fraction analyzed.

### 6.3. PGPR and Fe Acquisition

Rice-specific evidence should be interpreted according to the measured endpoint. Microbial modulation of phytosiderophore-related responses and Fe-acquisition pathways provides mechanistic evidence in rice [37]; the precise genes and their responses should be evaluated according to the measurements reported therein. However, direct, reproducible enrichment of edible grain—and particularly polished endosperm—requires separate validation. Studies reporting improved growth and yield under reduced N [39,40] cannot substitute for those measurements.

### 6.4. Nitrogen–Iron Integration

Integrating plant growth-promoting rhizobacteria (PGPR) with rational nitrogen fertilization management is a promising approach to maintain crop performance under optimized nitrogen fertilization while supporting physiological conditions favorable to iron nutrition in rice. Nitrogen is a major determinant of growth and yield, but its over-application reduces use efficiency and increases environmental losses; therefore, strategies that maintain productivity with lower inputs are particularly relevant for sustainable agriculture [39,40]. PGPR may support more efficient nitrogen management through biological nitrogen fixation, phytohormone production, stimulation of root growth, and improved nutrient acquisition; however, reduced-N performance should not be interpreted as direct evidence of increased nitrogen use efficiency unless that parameter is specifically quantified. In rice, studies with bacterial consortia and endophyte co-inoculation have shown that it is possible to maintain or improve growth and yield variables under reduced N doses, although the magnitude of the response depends on the environment, the strain, and agronomic management [39,40,60]. The nitrogen-to-iron relationship adds an important physiological dimension to this integration. Recent studies show that the homeostasis of both nutrients is coordinated by signaling networks that include NIN-like protein (NLP) factors, which are capable of linking N perception with genes involved in Fe acquisition, growth, and yield, particularly in rice [52,90]. Consequently, a balanced N supply can modify the plant’s response to Fe and should be considered when designing biofortification strategies. In this context, the combination of PGPR and nitrogen fertilization should be considered an integrated management strategy and not an automatic replacement for fertilizer. Selecting efficient strains, optimizing the N dose, and validating their performance across environments could help maintain crop productivity with optimized N inputs while simultaneously determining whether inoculation affects iron accumulation in the grain [40,90].

## 7. From Rhizosphere to Food Quality

### 7.1. Grain Fe Concentration

An increased iron concentration in the edible grain provides direct evidence of compositional biofortification, but it alone does not establish the nutritional value of the food. The available evidence indicates that increased iron in the rhizosphere can promote uptake by the plant, but the final accumulation in the grain depends on the combined efficiency of absorption, transport, remobilization, and tissue partitioning [49,53,56]. Therefore, microbial biofortification studies should quantify iron in roots, leaves, and reproductive organs, in addition to the grain, to identify the stage at which the main bottleneck occurs.

### 7.2. Fe Localization

The location of iron (Fe) within the grain is a critical component of nutritional quality. In rice, processing that removes the bran and aleurone layer reduces a significant portion of the mineral content, while the polished endosperm has a lower concentration of Fe [16,56,84]. Therefore, a comprehensive assessment must determine not only the total concentration but also the spatial distribution of Fe among the grain’s tissues and compartments. Location is relevant because it determines what fraction of the micronutrient remains after processing and what proportion is potentially available to the consumer.

### 7.3. Fe Bioaccessibility and Bioavailability

Bioavailability determines what proportion of ingested iron can be absorbed and used by the body. In plant-based foods, phytate is one of the main inhibitors of non-heme iron absorption due to its ability to form poorly soluble complexes with minerals; therefore, total iron content must be interpreted in conjunction with the composition of the food matrix [22,91]. Other dietary components also modify iron absorption. Polyphenols can reduce it by complexing the mineral, while ascorbic acid promotes the absorption of non-heme iron by keeping it in more soluble forms and reducing Fe^3+^ to Fe^2+^. The effect of calcium is more dependent on the dietary context and should not be considered a universal inhibitor of the same magnitude as phytates or polyphenols [4,22]. The microbiome introduces an additional dimension to the relationship between iron and nutrition. In the rhizosphere, microorganisms can modify the chemical availability of iron and activate plant mechanisms of acquisition and transport, influencing the iron that ultimately reaches edible tissues [27,49]. In the human body, iron participates in bidirectional interactions with the gut microbiome: iron availability can affect microbial community structure, while microbiota-derived molecules and competition for iron can influence host iron homeostasis [92]. The broader soil–plant–human gut microbiome axis is a useful conceptual framework, although direct causal evidence across the entire continuum remains limited [93]. Consequently, biofortification strategies aimed at human health should comprehensively assess the iron concentration in food and the factors that determine its biological utilization. Higher iron content in grain can increase the potential intake of this micronutrient, but the magnitude of the nutritional response will depend on its chemical form, the food matrix, processing, intestinal absorption, and the consumer’s physiological state [4,8,92].

Enhanced Fe mobilization is not uniformly beneficial. Under strongly reducing flooded conditions, elevated soluble Fe^2+^ can exceed physiological requirements and contribute to oxidative stress, bronzing, impaired root function, and yield losses in susceptible rice genotypes. Inoculant selection should therefore consider soil redox conditions, Fe toxicity risk, genotype tolerance, and water-management practices; maximizing soluble Fe is not an appropriate stand-alone performance target [46,85,86,87,88].

Therefore, nutritional evidence should be interpreted hierarchically. Consistent with Figure 2, the continuum can be organized into four sequential evidence levels: rhizosphere–plant Fe interactions; Fe accumulation in the edible grain; processing, cooking, and dietary bioaccessibility; and intestinal absorption and nutritional relevance. An increase in iron in the grain may increase the potential intake of the micronutrient, but does not automatically imply a proportional increase in intestinal absorption [4,8,22,92]. Demonstrating a nutritional benefit therefore requires specific studies of digestion, absorption, or nutritional efficacy and should not be inferred solely from chemical analyses of the grain. Figure 2 emphasizes two major translational bottlenecks across this continuum: transfer and partitioning of Fe into the edible grain, and conversion of grain Fe into bioavailable Fe.

## 8. Challenges and Future Perspectives

### 8.1. Context Dependency

A microbial strain that mobilizes Fe in vitro may fail to increase grain Fe under field conditions. Its performance depends jointly on plant genotype and Fe transporter regulation, soil pH and mineralogy, background Fe availability, water management and redox status, N fertilization, competition with resident microbiota, and inoculant establishment. Excessive Fe^2+^ mobilization in reduced soils may also increase toxicity risk; therefore, future trials should jointly monitor plant symptoms, growth, Fe speciation, and grain Fe endpoints across contrasting environments.

The effectiveness of plant–microorganism interactions is highly context-dependent. The plant genotype, identity and function of the microbial strain or community, pH, soil texture and mineralogy, iron availability, water regime, temperature, fertilization, and resident microbiome can all modify the response [52,60,74]. This dependence explains some of the variability between controlled studies and field conditions and constitutes one of the main challenges in transforming promising mechanisms into reproducible technologies.

The interaction between inoculants and native microbiota deserves special attention. Colonization, persistence, and functional expression of the inoculant depend on its ability to compete with or integrate into resident communities, while the effects of the inoculant on microbial networks can vary spatially and temporally [75,80,81]. Therefore, the selection of microorganisms should consider not only their in vitro siderophore-producing activity but also their compatibility with the host and their performance within the native community.

### 8.2. Field-Scale Validation

One of the main challenges in applying PGPR is translating results obtained in the laboratory, hydroponics, or greenhouse conditions to real-world agricultural systems. In the field, variations in soil, climate, management practices, genotype, and resident microbiome can alter inoculant survival and crop response, meaning that the efficacy observed under controlled conditions is not always consistently reproduced [74,75]. Spatial and temporal variability also influence the colonization and functional activity of the introduced microorganisms. Interaction with native communities can either promote or limit their establishment, and the final effect depends on the inoculant’s ability to occupy specific niches and maintain the desired function throughout the crop cycle [74,81]. These considerations justify conducting multi-site and multi-season trials before recommending a bioinput for widespread use. Adoption by farmers adds a technological and socioeconomic dimension. The availability of quality products, cost, ease of application, technical information, and, especially, the consistency of results influence the decision to use them. Therefore, scaling up requires integrating agronomic research, quality control, extension, and regulatory frameworks that allow for building trust and local evidence on the performance of inoculants [24,74].

### 8.3. Microbiome Engineering and SynComs

The development of synthetic microbiomes or synthetic communities (SynComs) offers a tool to reduce the complexity of natural communities and study defined combinations of microorganisms with complementary functions. These systems allow for the evaluation of causal interactions and the design of consortia with greater reproducibility; however, their stability, transferability to the field, and ability to compete with native microbiomes remain key challenges [94]. The selection of consortia should prioritize functional complementarity, ecological stability, and evidence of effective Fe transfer to the crop and, ultimately, to the edible fraction.

For translation into a deployable microbial product, candidate strains should be screened for identity, biosafety, genetic and phenotypic stability, siderophore activity under relevant soil conditions, carrier compatibility, viable cell counts at the end of shelf life, and establishment after seed or nursery application. Product claims should distinguish yield support from verified Fe enrichment in polished grain and, separately, demonstrate bioaccessibility or nutritional benefits. Multi-location field trials, batch quality control, dose optimization, and regulatory compliance are essential before commercialization.

### 8.4. Multiomics and Isotope Tracing

Omics technologies—genomics, transcriptomics, proteomics, metabolomics, and metagenomics—allow the correlation of microbial composition with gene expression, metabolites, and host physiological responses. Multi-omics integration with functional experiments can help identify genes, metabolites, and pathways associated with Fe acquisition and partitioning, and thus guide the rational design of biofortification strategies [83].

An additional methodological priority is the use of isotopic tracers, particularly stable Fe isotopes, to distinguish between mobilization, acquisition, translocation, and accumulation. These approaches would allow the establishment of transfer balances and the determination of whether the Fe mobilized by microorganisms actually reaches the harvestable organ, overcoming the limitations of inferring causality solely from total concentrations [83].

### 8.5. Formulation and Deployment

The formulation and deployment of bioinputs require maintaining the viability and function of microorganisms from production to application. Encapsulation, protective carriers, and consortia can improve stability and release, but compatibility between microorganisms, shelf life, rhizosphere persistence, ease of application, and performance under real-world agricultural conditions must be evaluated [82]. Adoption also depends on cost, the availability of quality products, ease of use, quality control, and consistency of results. Therefore, scaling up must integrate agronomic research, formulation, multi-site validation, extension, and regulatory frameworks.

### 8.6. Integrated Limitations and Evidence Priorities

The current evidence base is heterogeneous in intervention type, crop, cultivation system, and analytical endpoint. Therefore, findings from purified siderophore applications, intercropping, and water-management comparators should not be pooled conceptually with controlled microbial inoculation trials as if they establish the same causal pathway. The grouped evidence in Table 1 is illustrative rather than exhaustive, and its qualitative hierarchy is not a formal risk-of-bias assessment.

For a credible causal test in rice, future experiments should identify the inoculant at strain level, report its application method and persistence, compare inoculated and uninoculated controls under equivalent Fe and N inputs, and document soil pH, redox potential, irrigation regime, genotype, and background microbiota. Outcomes should include yield, grain Fe concentration, and Fe mass per area, and—when feasible—separate brown and polished grain fractions. Isotope tracing or other validated approaches are also needed to distinguish Fe mobilized by microbial siderophores from Fe entering through conventional plant uptake routes.

The nutritional inference should be evaluated independently of agronomic response. Greater total Fe in harvested grain solely represents evidence of compositional enrichment; evidence of retention after milling, simulated-digestion bioaccessibility, intestinal absorption, and nutritional efficacy requires distinct measurements. In flooded rice, interventions that increase soluble Fe^2+^ must also be evaluated for potential Fe toxicity, genotype-dependent tolerance, and unintended effects on other elements.

## 9. Conclusions

The available evidence indicates that plant–microorganism interactions can play a role in different stages of plant iron nutrition, particularly through the production of siderophores, the modification of rhizosphere chemistry, iron mobilization, and the modulation of physiological responses associated with its acquisition and homeostasis. However, evidence demonstrating consistent and reproducible increases in iron in the edible fraction of crops, and especially improvements in its bioaccessibility or bioavailability, remains limited. Therefore, the main challenge lies not only in increasing iron availability in the rhizosphere, but also in demonstrating that this increase translates into greater uptake by the plant, efficient translocation to the harvestable organ, and nutritionally relevant accumulation in the grain.

Rice is a priority system due to its importance as a global staple food, the low iron density in the polished grain, and the accumulated knowledge of the mechanisms of acquisition, transport, and homeostasis of this micronutrient. Integrating beneficial microorganisms with balanced nitrogen fertilization management represents a potential strategy to maintain crop performance under optimized N inputs while promoting physiological conditions associated with iron nutrition; improvements in nitrogen use efficiency should be claimed only when they are directly quantified. However, increased iron availability in the rhizosphere or a positive response to the inoculant should not be automatically interpreted as evidence of biofortification, since iron accumulation in the grain depends on the interaction between nutrient availability, the plant’s acquisition and translocation capacity, tissue partitioning, and environmental conditions.

To move from proof of concept to reproducible agronomic applications, multi-site and multi-season trials are required to evaluate the stability of responses under different soil and environmental conditions, as well as to characterize the interaction between inoculants and the native microbiome. Furthermore, it is necessary to develop stable microbial formulations and employ omics, physiological, and nutrient-tracing tools to elucidate the causal mechanisms linking microbial activity to iron acquisition and partitioning. The evaluation should extend to the nutritional quality of the food, considering iron concentration and distribution in the grain, processing-related losses, bioaccessibility, and bioavailability. In this context, plant–microbe biomanufacturing can be considered a complementary framework to genetic and agronomic biofortification, with biofabrication serving as its operational concept for coordinating microbial functions and plant physiological processes toward improved iron nutrition. Its contribution to nutritional security will ultimately depend on sequentially demonstrating micronutrient transfer to edible tissues, retention during processing, and subsequent bioaccessibility or bioavailability.

## Figures and Tables

**Table 1:** Representative evidence arranged by demonstrated endpoint and their relevance to microbiome-assisted Fe biofortification. Group A: direct grain/seed Fe evidence following microbial or microbially derived interventions; Group B: mechanistic/supporting evidence without demonstrated edible-grain enrichment; Group C: agronomic or environmental contextual evidence (not direct microbial Fe biofortification). NR, not reported or not directly evaluated. Study types are heterogeneous and are not ranked by effect size.

Main Evidence/ Process	Crop/ Intervention	Siderophore or Fe Mobilization	Growth/ Yield	Tissue Fe	Grain/ Seed Fe	Processed Grain Fe/ Bioaccessibility/ Bioavailability	Ref.
GROUP A PGPR-mediated grain Fe enrichment	Rice; PGPR field inoculation	Not quantified as endpoint	Yes	Yes	Yes; selected PGPR nearly doubled grain Fe	NR	[31]
GROUP A Fe solubilization and grain Fe enrichment	Rice; Fe-solubilizing PGPB, field	Yes; Fe solubilization	Yes	Yes	Yes; reported 53.88–89.05% increase in grain Fe	NR	[32]
GROUP A Microbial-assisted grain Fe biofortification	Wheat; endophytic Bacillus altitudinis WR10	Yes; siderophore-producing strain	Yes	Yes	Yes	NR	[33]
GROUP A Siderophore-mediated grain Fe enrichment	Wheat/soybean; Fe-fortified bacteriosiderophore foliar spray	Yes; purified bacterial siderophore	Yes	Yes	Yes; ~2.0-fold wheat, 1.7-fold soybean	NR	[34]
GROUP A Endophyte-assisted grain Fe enrichment	Quinoa; B. phytofirmans PsJN + biochar	Yes; siderophore-producing endophyte	Yes	Yes	Yes; up to 71% increase	NR	[35]
GROUP A Consortium-assisted grain Fe enrichment	Sorghum; Bacillus consortium	Siderophore/PGP traits	Yes	NR	Yes; up to 97% increase	NR	[36]
GROUP B Microbial induction of Fe-acquisition responses	Rice; P. simiae WCS417 + D. hansenii	Phytosiderophore response/Fe-acquisition genes	Growth/physiology	Fe-acquisition response	NR	NR	[37]
GROUP B Rhizobacterial enhancement of Fe nutrition	Maize–peanut intercropping; siderophore-secreting rhizobacteria	Yes	Yes	Yes/Fe nutrition	Edible-organ Fe evidence not the primary endpoint	NR	[38]
GROUP C Growth/yield response under reduced N	Rice; bacterial consortium + reduced N	Not an Fe endpoint	Yes	NR	NR	NR	[39]
GROUP C Growth response under reduced N	Rice; Bacillus/Priestia + reduced N	Not an Fe endpoint	Yes	NR	NR	NR	[40]
GROUP C Water/redox effects on grain Fe	Rice; AWD vs. continuous flooding	No microbial inoculant; water/redox comparator	Yes	Elemental composition	Yes; AWD altered/decreased grain Fe depending on site	NR	[41]

## Data Availability

No new data were created or analyzed in this study. Data sharing is not applicable to this article.

## Abbreviations

The following abbreviations are used in this manuscript:

Fe	Iron
N	Nitrogen
PGPR	Plant growth-promoting rhizobacteria
ISR	Induced systemic resistance
NA	Nicotianamine
IRT	Iron-regulated transporter
YSL	Yellow Stripe-Like transporter
NLP	NIN-like protein
SynComs	Synthetic microbial communities
